# Vector representation based on a supervised codebook for Nepali documents classification

**DOI:** 10.7717/peerj-cs.412

**Published:** 2021-03-03

**Authors:** Chiranjibi Sitaula, Anish Basnet, Sunil Aryal

**Affiliations:** 1 Deakin University, Geelong, VIC, Australia; 2 Ambition College, Kathmandu, Nepal

**Keywords:** Text classification, Machine learning, Codebook, Nepali documents, Classification, Feature extraction

## Abstract

Document representation with outlier tokens exacerbates the classification performance due to the uncertain orientation of such tokens. Most existing document representation methods in different languages including Nepali mostly ignore the strategies to filter them out from documents before learning their representations. In this article, we propose a novel document representation method based on a supervised codebook to represent the Nepali documents, where our codebook contains only semantic tokens without outliers. Our codebook is domain-specific as it is based on tokens in a given corpus that have higher similarities with the class labels in the corpus. Our method adopts a simple yet prominent representation method for each word, called probability-based word embedding. To show the efficacy of our method, we evaluate its performance in the document classification task using Support Vector Machine and validate against widely used document representation methods such as Bag of Words, Latent Dirichlet allocation, Long Short-Term Memory, Word2Vec, Bidirectional Encoder Representations from Transformers and so on, using four Nepali text datasets (we denote them shortly as A1, A2, A3 and A4). The experimental results show that our method produces state-of-the-art classification performance (77.46% accuracy on A1, 67.53% accuracy on A2, 80.54% accuracy on A3 and 89.58% accuracy on A4) compared to the widely used existing document representation methods. It yields the best classification accuracy on three datasets (A1, A2 and A3) and a comparable accuracy on the fourth dataset (A4). Furthermore, we introduce the largest Nepali document dataset (A4), called NepaliLinguistic dataset, to the linguistic community.

## Introduction

We need to represent documents mathematically to perform machine learning tasks such as classification, clustering and so on. Text documents are represented by using words (tokens) present in them. Because of the rise of social media and scattered news online, an automated document classification has been an important research domain to predict the trending news online automatically. Recently, there have been several works (Mourão et al., 2018; Kim et al., 2019; Elnagar, Al-Debsi & Einea, 2020; Shan et al., 2020; Silva et al., 2020) in document representation and classification, especially in non-Nepali language domains such as English, Portuguese, Arabic, and so on. However, very few works (Subba, Paudel & Shahi, 2019; Singh, 2018; Dangol, Shrestha & Timalsina, 2018; Basnet & Timalsina, 2018; Kafle et al., 2016; Thakur & Singh, 2014; Shahi & Pant, 2018) have been carried out in the Nepali document representation and classification, where Nepali documents are based on Devanagari alphabets, which has 36 consonants, 13 vowels and 10 numerals of Nepali language (see details in Table 1). Devanagari alphabets and their variants, letters and special characters are provided in Section 4 of the Supplemental File. Devanagari alphabets do not have capital letters and are written left-to-right order horizontally. With the prolific growth of Nepali documents online in Nepal and abroad, it has opened up numerous avenues for the automatic processing (e.g., classification) of such documents.

**Table 1:** Nepali numerals, consonants and vowels.

Numerals	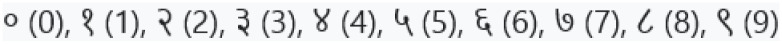
**Consonants**	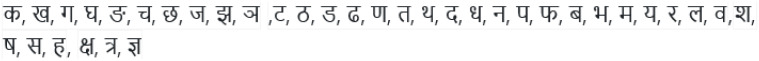
**Vowels**	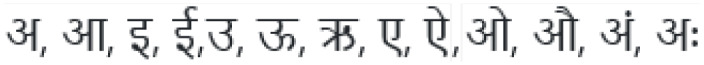

While reviewing existing works in literature for both Nepali and non-Nepali document representation and classification, we notice that traditional machine learning-based and deep learning-based methods have been frequently adopted. Traditional machine learning algorithms adopt different techniques such as Vector Space Models, N-gram models, Latent Dirichlet Allocation (LDA), etc. Similarly, deep learning algorithms employ several methods, such as Convolution Neural Networks (CNNs), Long Short-Term Memory (LSTM), Capsule Networks (CapsNet), etc. CNNs have also been widely used in different areas apart from text classification, such as scene image analysis (Sitaula, 2013), COVID-19 chest x-ray image analysis (Das, Santosh & Pal, 2020; Sitaula & Hossain, 2020; Mukherjee et al., 2020), breast image analysis (Sitaula & Aryal, 2020), Devanagari script analysis (Guha et al., 2020), Computational offloading (Khayyat et al., 2020), Input validation (Wang et al., 2020a), Kubernetes cluster (Mao et al., 2020), etc. Nevertheless, popular deep learning-based algorithms (e.g., CNN (Elnagar, Al-Debsi & Einea, 2020), LSTM (Wang et al., 2020b), CapsNet (Kim et al., 2020), etc.) that have been used in the representation and classification of documents do not utilize any outlier tokens elimination strategies. Outliers or contaminated tokens could compromise the classification accuracy despite the proven efficacy of different state-of-the-art methods in several other domains.

Outlier tokens are attributed to the performance loss during classification, which is a common problem in document classification including Nepali documents. Existing document representation methods are mostly based on syntactical approaches such as Term Frequency-Inverse Document Frequency (TF-IDF) (Shahi & Pant, 2018), Bag of Words (BoW) (Salton & McGill, 1986), which consider the Term Frequency (TF) and Inverse Document Frequency (IDF) to weight each token present in the document. Nevertheless, such methods still lack the strategy to eliminate the outliers, which exacerbate the classification performance in the end (Aryal et al., 2015, 2019). To mitigate such problems, we propose to use the supervised codebook, which is also called a domain-specific filter bank and has been used in some of the existing methods (Sitaula et al., 2019a, 2020) to filter out unimportant tokens. Since such a codebook only captures the domain-specific tokens and ignores the irrelevant tokens, we will be able to achieve more robust features. However, such existing codebook methods still have three main limitations: (i) they only rely on cosine similarity of tokens with the pre-defined labels or categories merely, which ignore the semantic relationship of tokens that could be very important within the documents; (ii) they ignore the semantic relationship of categories under the domain of study, which could be an interesting clue to achieve the resultant codebook; and (iii) they use a hard threshold, which could miss the important discriminating tokens having lower threshold during supervised codebook design. Besides, we do not have a state-of-the-art pre-trained word embedding model for Nepali words, as in English (Mikolov et al., 2013; Pennington, Socher & Manning, 2014; Bojanowski et al., 2017), that can be used to capture the semantic association of tokens.

**Figure 1:**
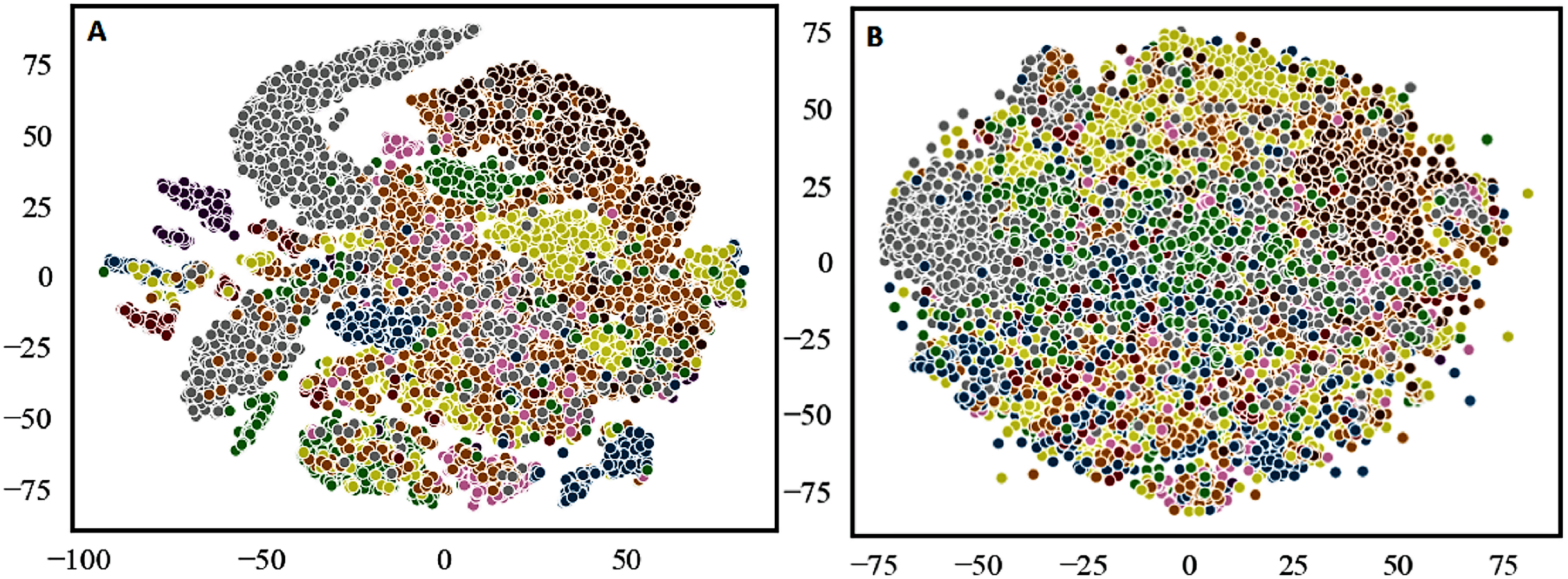
The t-SNE (t-distributed Stochastic Neighbor Embedding) scatter plots (Maaten & Hinton, 2008) of: (A) our proposed method; and (B) TF-IDF method, on the training set of Combined Nepali News dataset (Set 5), where the different colours represent different categories.

To address the above mentioned gaps in the existing supervised codebook, we adopt the following strategies. First, to show the semantic association of tokens in a document, we track the occurrences of neighboring tokens of the category labels in the document. It plays a crucial role to extract semantically related tokens only and discards irrelevant tokens. Second, to show the semantic relationship of categories, we calculate the cosine similarity of the token with all the categories, thereby finding the best candidate category for the input token. Last, use of both steps (i) and (ii) to eliminate the hard threshold criteria and the resulting features improve the discriminability (see the visual comparison of the t-distributed Stochastic Neighbor Embedding (t-SNE) (Maaten & Hinton, 2008) scatter plots using our supervised codebook-based method against TF-IDF method in Fig. 1). Furthermore, for each token to be used in the experiment, we propose to use the probability-based embedding to capture the semantic relationships of tokens with category labels.

To design our proposed codebook, we perform the following steps. First, we propose to use the probability-based word embedding of each token based on the training dataset. Next, we design a supervised codebook using category labels and tokens of documents under all categories in the training dataset. For this, we consider the semantic relatedness of neighboring tokens using cosine similarity. Note that semantic relatedness of tokens is captured based on both the corresponding category and other remaining categories. To obtain the representation for a given document based on such codebook, we calculate the cosine similarity of each token of the document with all tokens of the codebook, which results in a matrix. Last, we take the column-wise average of the matrix to obtain the feature vector representing the input document, whose size is equal to the codebook length.

The main **contributions** of this article are as follows:

- We develop a novel approach to represent the Nepali new documents for the classification purpose.
- We release a new large Nepali news collection with 35,651 documents divided into 17 categories.
- We evaluate our method on four Nepali news classification datasets using the Support Vector Machine (SVM) classifier and compare with state-of-the-art methods, including traditional methods (for example, TF-IDF, LDA, etc.) and Deep Learning (DL)-based methods (for example, Word2vec, LSTM and Bidirectional Encoder Representations from Transformers (BERT)). The evaluation results show that our method provides a stable and consistent performance compared to state-of-the-art methods.

The rest of the article is organized as follows. We review key related works in the Nepali and non-Nepali news classification task in the next section (“Related Works”) and then present our new Nepal news collection dataset (“NepaliLinguistic Dataset”) and our proposed new Nepali document representation method (“Proposed Method”) in the next two sections. We discuss our experimental results in the second last section (“Experiments and Analysis”) before concluding the article with potential future work in the last section (“Conclusion and Future Works”).

## Related works

In this section, we review some recent state-of-the-art methods used to represent and classify news or text documents in different languages. Therefore, we divide the whole section into two subsections: Nepali and non-Nepali news document representation methods.

### Nepali news document representation methods

There have been a very few works (Subba, Paudel & Shahi, 2019; Singh, 2018; Dangol, Shrestha & Timalsina, 2018; Basnet & Timalsina, 2018; Kafle et al., 2016; Thakur & Singh, 2014; Shahi & Pant, 2018) performed in Nepali news document representation for the classification purpose. Thakur & Singh (2014) used BoW for the representation of the Nepali news documents and classified them using the Lexicon pooling approach based on Naive Bayes algorithm. Kafle et al. (2016) performed a comparative study of two different document representation methods, including TF-IDF and word2vec embedding-based method (Mikolov et al., 2013) on Nepali news classification. Singh (2018) used TF-IDF as the representation of Nepali texts that are achieved from books, newspapers, journals, etc. and classified separately using different algorithms such as Logistic Regression, SVM, Multinomial Naive Bayes, Bernoulli Naive Bayes, Nearest Neighbor and so on. Shahi & Pant (2018) used TF-IDF method to achieve features of Nepali news documents and classified using Naive Bayes (Lewis, 1998), SVM (Cristianini & Shawe-Taylor, 2000) and Neural Networks. Dangol, Shrestha & Timalsina (2018) used the n-gram model (Brown et al., 1992) of texts in the news documents and designed term-document matrix based on it for the classification purpose. Basnet & Timalsina (2018) extracted features based on the word2vec model (Mikolov et al., 2013) and performed classification using the LSTM Neural Network model. Subba, Paudel & Shahi (2019) used BoW features (Salton & McGill, 1986) of the Nepali news articles to train the deep learning network.

In summary, most of the works in Nepali news representation methods are based on BoW and TF-IDF methods, which calculate the weights of tokens based on the syntactic approach. However, such methods are unable to work accurately for documents containing out-of-vocabulary tokens in the document. Similarly, the relationship between words present in a document could further provide the semantic meanings, which has also been ignored in literature. To fulfill such gaps in Nepali news document representation, we propose a novel method that captures the semantics of tokens present in the document to yield better differentiation.

### Non-Nepali news document representation methods

We review some of the recent methods (Mourão et al., 2018; Kim et al., 2019; Elnagar, Al-Debsi & Einea, 2020; Shan et al., 2020; Silva et al., 2020) to represent and classify news documents in other languages such as English, Portuguese and so on. Mourão et al. (2018) proposed a novel method, called Net-Class, to represent and classify the news documents in English language. The relationship of words in documents are extracted using graph theory. Kim et al. (2019) proposed a new model, called multi co-training, using three representation methods: TF-IDF, LDA and Document to Vector on English news documents. Their method outperforms each method in the classification task. Elnagar, Al-Debsi & Einea (2020) used a deep learning model to categorize the Arabic news documents. They used Recurrent Neural Networks and CNNs for features extraction and classification purposes. Shan et al. (2020) proposed an incremental learning strategy based on a deep learning approach to represent and classify English news documents. Silva et al. (2020) performed Portuguese news documents classification to capture fake news. They used BoW to represent the documents in their work. Faustini & Covões (2020) adopted BoW and Word2Vec models (Mikolov et al., 2013) to represent and classify documents for fake news detection in various languages such as German, Latin, Slavic, etc. Kim et al. (2020) represented and classified news documents using capsule networks (Sabour, Frosst & Hinton, 2017). Wang et al. (2020b) used Convolutional Neural Network and Bidirectional Long Short-Term Memory (CNN–BiLSTM) for the representation and classification of Chinese news classification tasks.

While analyzing previous works in the literature for both Nepali and non-Nepali document classification, we notice that two kinds of techniques, deep learning (DL)-based and traditional BoW-based methods, have been extensively adopted. Nonetheless, both kinds of techniques may not be appropriate to Nepali documents representation because of two reasons. First, such methods lack the strategies to eliminate outlier tokens in the documents and need a massive amount of data for training. Second, traditional methods, which mostly focus on syntactical approaches, may not be suitable for our datasets due to the presence of numerous outlier tokens in the documents. The advantages and disadvantages of existing methods discussed in literature are presented Section 2 of the Supplemental File.

## Nepalilinguistic dataset

In this section, we present our new Nepali news dataset. To design the dataset, we crawl news documents for each category from three popular online news portals including Kantipur online (https://ekantipur.com/), Ratopati (http://ratopati.com/) and Nagarik News (https://nagariknews.nagariknetwork.com/) from 20 February 2020 to 18 March 2020. We choose these three websites because they are the leading online news media in Nepal, among which ekantipur is the most popular.

There are 17 news categories, which includes Art, Bank, Blog, Business, Diaspora, Entertainment, Filmy, Health, Hollywood-bollywood, Koseli, Literature, Music, National, Opinion, Society, Sports and World. There are 35,651 documents in the dataset with the Sports category containing the highest number of documents and tokens and the Health category containing the least number of documents and tokens. The dataset is available publicly at (https://ieee-dataport.org/documents/nepaliliinguistic) for researchers to use in their research. The detailed information of the dataset is available in Section 3 of the Supplemental File.

## Proposed method

**Figure 2:**
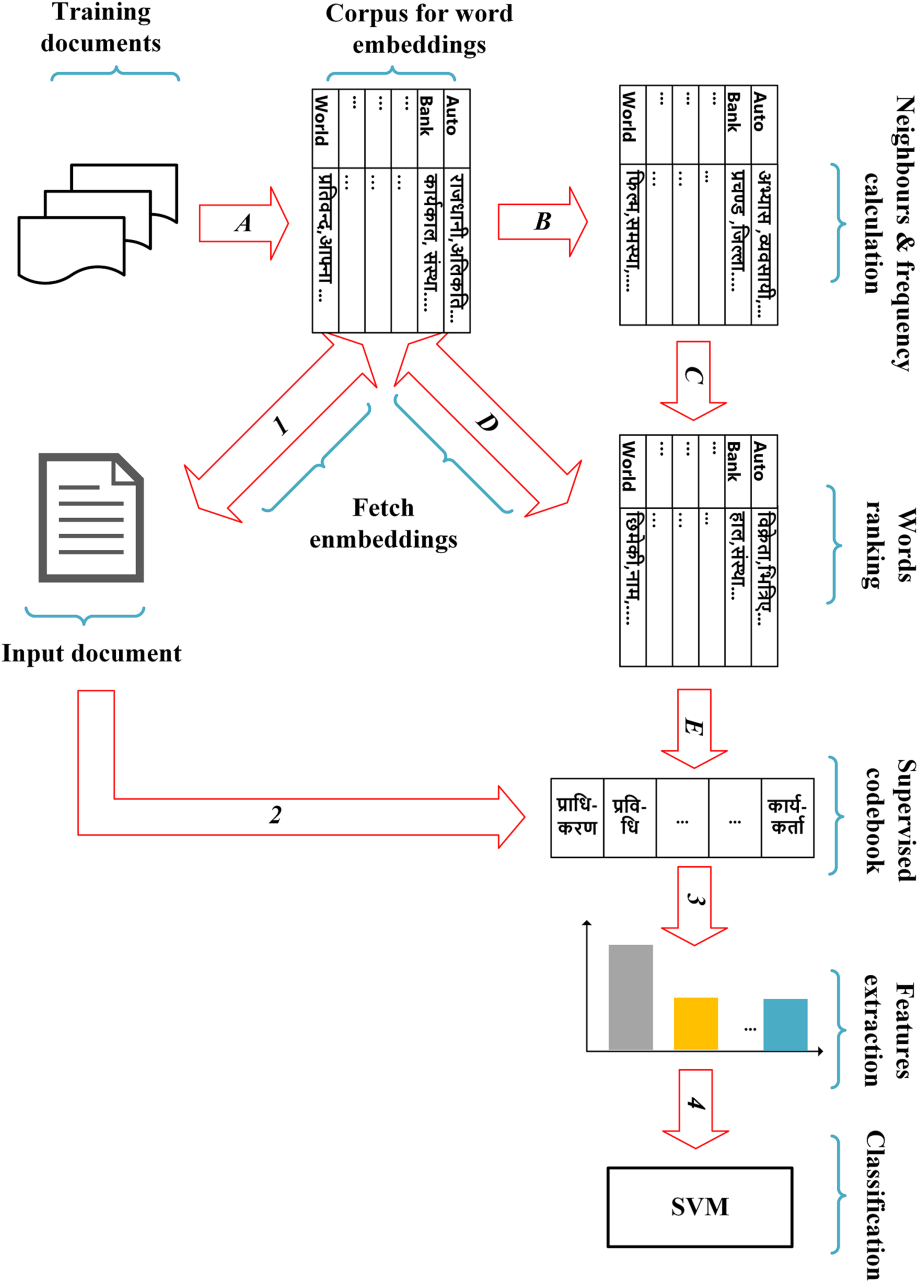
The overall pipeline of the proposed method. Note that the steps A, B, C, D and E are performed using training documents to achieve the supervised codebook, whereas the steps 1, 2, 3 and 4 are used to achieve our proposed features of each input document based on the codebook learned from the training documents.

Our proposed method has four main steps: pre-processing of documents, extraction of probability-based word embedding vector for each pre-processed token of the documents, design of a supervised codebook and feature extraction. The overall pipeline of our method is shown in Fig. 2.

### Pre-processing of documents

We pre-process each news document using different techniques including tokenization, alphanumeric characters removal, stop words removal, stemming operations and unique words extraction. First, we perform tokenization and alphanumeric character removal. For tokenization and alphanumeric character removal, we use Natural Language Toolkit (NLTK) (https://www.nltk.org/), which uses white space characters to tokenize words or tokens from the sentence and has a pre-defined alphanumeric character list for the elimination of alphanumeric characters for the input text documents. Next, we perform stemming of Nepali words, which is complex (Sitaula, 2013; Bal & Shrestha, 2004; Bal, 2009; Paul, Dey & Purkayastha, 2014; Prabha et al., 2018) in most cases due to variable structures. For this, we use a simple Nepali stemmer (https://github.com/sanjaalcorps/NepaliStemmer), which is publicly available. Next, we remove stop words present in the documents (refer to Section 4 of the Supplemental File). Since there is no such well-established stop words list available in the Nepali linguistic community due to the variability in Nepali writing, we prepare a pre-defined list of stop words, which are the list of unimportant tokens in the documents, and apply them by the rule-based method in the documents to remove such words. We use string matching technique in the rule-based method to filter out stop words from documents. We consider only those words that do not belong to a pre-defined list of stop words and discard those words belonging to the list. Last, people write the same Nepali words or tokens differently. This is because of the presence of similar letters or alphabets that can be used interchangeably. Given this information, we prepare a pre-defined list of special alphabets to achieve the unique tokens. Please refer to Section 4 of the Supplemental File for the examples of stop words, pre-defined list of special alphabets and pre-processed text after all aforementioned operations.

### Extract word embedding of each pre-processed token in the documents

Natural Language Processing (NLP) research in Nepali language lacks the state-of-the-art word embedding techniques such as Word2vec (Mikolov et al., 2013), GloVe (Pennington, Socher & Manning, 2014) and fastText (Bojanowski et al., 2017) available in other languages. So, we propose our probability-based word embedding technique. Our idea is motivated by the fact that the importance of a token, which can be shown by probability-based technique, in different categories of training set provides its semantic meanings (Sitaula et al., 2019b). For each category *i*, we create a single document file *D*_*i*_ by appending all documents in the training collection belonging to the category. Then, the probability of each token in each category is computed from category-based aggregated single files resulting in an embedding vector for the token of the length equal to the number of categories (refer to Eq. (1))

(1) $$\{ t(i)\} _{i = 0}^p = \displaystyle{{\lambda (t|{D^i})} \over {\lambda ({D^i})}}$$

In Eq. (1), let us assume that *t* is the input token and $\{ t(i)\} _{i = 0}^p = \{ t(0),t(1), \cdots ,t(p)\}$ represents its embedding vector in Eq. (1). Here, λ(*t*|*D*^i^) represents the count of token *t* in *D*^i^, whereas λ(*D*^i^) is sum of the counts all tokens in *D*^i^. As a result, if we have *p* categories in the dataset, the resultant embedding vector size will be *p*-D for each token. Examples of sample embedding vectors of three tokens are presented in Section 4 of the Supplemental File.

### Design of the supervised codebook

After the extraction of the embedding vector of each token, our next step is to design a supervised codebook using the training set only. It is the most important step in our method. We follow the following five steps. First, we search all the neighboring tokens using the left and right index positions of each input token of the corresponding category label within its category documents. With the help of such neighboring tokens of the corresponding category, we capture its semantically related tokens only and discard other unimportant tokens. The selection of neighbors only might not always be sufficient to capture the most important tokens. For example, some unimportant tokens may be extracted because of the neighboring relations with the corresponding category label. So, we adopt another idea of token occurrence in the documents. We calculate the occurrence (frequency) of such neighboring tokens in the corresponding categories. The higher the occurrence of neighboring tokens, the more important tokens are for the corresponding category. We repeat such operation for all categories. However, our goal is to select the most appropriate tokens having higher frequencies. For this, we utilize occurrences of both the corresponding category label itself and the neighboring tokens. In each category, we compare the occurrences (frequencies) of neighboring tokens and the corresponding category label itself. If the occurrence of the token is higher than that of the category label, the token is selected, otherwise, it is discarded. Using this idea, for each category, we capture only those tokens that have higher frequencies than the category label itself because we believe that if a token is occurring higher than the corresponding category label, it shows the higher semantic association between them. We repeat such a process for all the categories and prepare the unique list of tokens for each category. However, from the previous step, the tokens in one category having the highest occurrences might still be more semantically related to another category. Next, we calculate the cosine similarity of the token with both the corresponding category and other remaining categories. If the cosine similarity of the token with the corresponding category label is higher than other category labels, it is accepted, otherwise, it is discarded. Note that the embedding vectors of tokens and category labels using our proposed embedding discussed above are used to compute the similarity. We repeat such a process for all categories. Last, we combine all the list of tokens from all categories to form a single list, which acts as our proposed supervised codebook to be used in our method. The example of our proposed supervised codebook and size of codebook on four datasets can be seen in Sections 4 and 5 of the Supplemental File, respectively.

The detailed step-wise procedure to achieve our codebook is presented in Section 6 of the Supplemental File. In the algorithm, *D* represents the collection of pre-processed corpus set ({*D*^1^, *D*^2^, ⋯, *D*^p^}) for the corresponding categories ({*C*^1^, *C*^2^, ⋯, *C*^p^}); *p* represents the total number of categories for the corresponding dataset and *D*^i^ represents the corpus, which is the concatenation of all the documents under the corresponding category (*C*^i^). In the meantime, we utilize cosine similarity (Eq. (2)) based on embedding vectors to show the semantic similarity between tokens.

(2) $$cos({k_1},{k_2}) = \displaystyle{{{k_1} \times {k_2}} \over {||k1|| \times ||{k_2}||}},$$

where *k*_1_ and *k*_2_ represent two tokens to be used for calculating their cosine similarity (cos(.)).

Likewise, the size of the codebook in each dataset depends on the number of tokens and categories.

### Feature extraction

This is the final step in our method, also called the feature extraction step, which is based on the supervised codebook. For this, we are motivated by Liu et al. (2017), who uses Resnik measure (Resnik, 1995) between concepts and words to construct a document matrix and then, perform sum aggregation across it to represent each document. Nevertheless, we use the cosine similarity measure with the average aggregation method, which is appropriate to our domain. For this, we calculate the cosine similarity of each input token of the document with all the codebook tokens to construct the document matrix for each document. At last, we average all the instances (or rows) of the matrix to achieve our final proposed features of the document. Note that the size of the proposed features is equal to the size of the codebook (i.e., |*F*|).

Specifically, if there are *n* words in the document and *m* (i.e., |*F*| = *m*) tokens in the codebook, we achieve the matrix of size *n* × *m* for each document. Then, we aggregate all the instances of the matrix using the average aggregation method to achieve our proposed feature vector of size *m*-D (Eq. (3)).

(3) $$\{ P({S_j})\} _{j = 0}^m = \displaystyle{{\sum\limits_{i = 0}^n T_j^i} \over n},$$

where $\{ P({S_i})\} _{i = 0}^m = \{ P({S_0}),P({S_1}), \cdots ,P({S_m})\}$ represents the aggregated feature vector of the input document *P*. Similarly, $\sum\nolimits_{j = 0}^n T_j^i$ provides the sum of all row elements (*n*) for *i*th column in the matrix *T*. Meanwhile, the detailed steps of our feature extraction method are also shown in Section 6 of the Supplemental File.

## Experiments and analysis

### Datasets

Since we are focusing on Nepali document classification, we utilize two publicly available datasets (16NepaliNews^1^ 1Information and Language Processing Research Lab, Kathmandu University, Nepal. and NepaliNewsLarge (Shahi & Pant, 2018)), the combination of such two datasets, and our new Nepali news dataset, called NepaliLinguistic, which we collected and presented in the article. In total, four datasets are taken for the evaluation of our method. For the train/test split of each category on each dataset, we randomly divide documents per category into 90%/10% ratio for the experiment. We perform such experiments in five-round and report the average performance. Further detailed descriptions of the four datasets are also provided in Section 7 of the Supplemental File

**16NepaliNews** contains 14,364 documents under 16 categories, where each category contains at least 16 documents.

**NepaliNewsLarge** contains 7,023 documents under 20 news categories, where each category contains 111 to 700 documents.

**CombinedNepaliNews** contains 21,387 documents under 21 categories, where each category contains 111 to 7,452 documents.

**NepaliLinguistic**, which is a new dataset we prepared and will be made publicly available, contains 17 news categories.

### Implementation

To implement our work, we use Python (Rossum, 1995) programming language, which is open source and has extensive support libraries, including Scikit-learn (Pedregosa et al., 2011). Similarly, to perform the classification, we use the SVM classifier (Cristianini & Shawe-Taylor, 2000), which is one of the popular classification algorithms in machine learning research (Fernández-Delgado et al., 2014). In SVM, we need to choose optimal parameters such as *gamma*, *kernel*, *C*, etc. depending on the nature of datasets. We set the *gamma* = 1*e* − 04 and *kernel* = *rbf* as default in the SVM classifier. We empirically set such default parameters before tuning *C* parameter, which tells SVM optimization how much we want to discard misclassifying each training example. To tune the *C* parameter automatically, we perform grid searching of *C* value in the range {1, 11 ,21, ⋯, 91} with 10 uniform steps during the classification. In the meantime, we perform our experiment on a machine with Intel core i5-6200U 2.30 GHZ CPU and 12 GB RAM.

### Comparison with state-of-the-art methods

We compare our method with both traditional methods and recent deep learning-based (DL-based) methods for the classification. For traditional methods, we implement some popular text feature extraction methods such as BoW (Salton & McGill, 1986), TF-IDF (Robertson, 2004) that has employed by Shahi et al. (Shahi & Pant, 2018) and LDA (Blei, Ng & Jordan, 2003). For LDA, we use 12 topics on all datasets, which we find empirically the best among different topics. From empirical study, we notice that the number of topics less than 12 deteriorates the classification performance because it may not be able to cover all the contexts of such documents. Also, the number of topics greater than 12 could further disintegrate the discriminating topics, thereby resulting in lower classification performance. Thus, we conjecture that each dataset used in our work has normally 12 topics, which helps to better differentiate them.

Furthermore, for the fair comparison of our method with the BoW and TF-IDF methods, we extract the features size equal to our codebook (*F*) size. For DL-based methods, we use recent deep learning-based methods for document representations such as Word2Vec (Kafle et al., 2016), LSTM (Basnet & Timalsina, 2018), and BERT (Devlin et al., 2018). For Word2Vec, we achieve a 300-D feature vector for each token. For LSTM, we use the optimal architecture as suggested by Basnet & Timalsina (2018), which is 300-150-Softmax architecture (first layer with 300 units, second layer with 150 units, which is followed by the Softmax layer). For the BERT, we leverage the pre-trained weights that has been prepared for multi-lingual domain (https://github.com/google-research/bert/blob/master/multilingual.md) and set all the parameters as default. The comparative results using classification accuracy of our method with state-of-the-art methods on four datasets (16NepaliNews, NepaliNewsLarge, CombinedNepaliNews, and NepaliLinguistics) are shown in Table 2. The table has five columns, where the first column lists the methods to be compared, second column lists the classification accuracies for 16NepaliNews, third column lists the classification accuracies for NepaliNewsLarge, forth column lists the classification accuracies for CombinedNepaliNews, and fifth column lists the classification accuracies for NepaliLinguistics datasets.

**Table 2:** Comparative analysis using classification accuracy (%) of our method with state-of-the-art methods on four datasets. Note that A1, A2, A3 and A4 denote 16NepaliNews, NepaliNewsLarge, CombinedNepaliNews and NepaliLinguistic, respectively. Best result is shown in bold face.

Methods	A1 (%)	A2 (%)	A3 (%)	A4 (%)
BoW (Salton & McGill, 1986)	73.48	51.08	67.73	92.43
LDA (Blei, Ng & Jordan, 2003)	66.77	39.34	54.52	78.29
BoW+boolean	74.00	54.44	68.64	**92.52**
TF-ICF (Wang & Zhang, 2010)	73.48	51.08	67.73	92.43
Word2Vec (Kafle et al., 2016)	74.57	51.11	64.98	89.63
LSTM (Basnet & Timalsina, 2018)	75.52	47.30	71.24	90.32
Shahi & Pant (2018)	73.48	51.08	67.73	92.23
BERT (Devlin et al., 2018)	75.08	58.16	69.63	88.88
Ours	**77.46**	**67.53**	**80.54**	89.58

In the second column of Table 2 for 16NepaliNews, we notice that our method outperforms all eight methods (five traditional and three recent DL-based methods) used in the experiments with the classification accuracy of **77.46%**. Specifically, our method imparts at least 1.94% higher than the second-best method (LSTM (Basnet & Timalsina, 2018)). Interestingly, our method also outperforms BERT (Devlin et al., 2018) with a margin of over 3.00% on such dataset. Furthermore, while looking in the third column of Table 2 for NepaliNewsLarge, we observe that our method outperforms all contender methods (five traditional and three recent DL-based methods) with a significant margin of classification accuracy (9.37%) against the second-best method (BERT (Devlin et al., 2018)). Our method is prominent on this dataset as well. Similarly, the fourth column of Table 2 for CombinedNepaliNews shows that our method again outperforms all existing eight methods with a significant margin of at least 10.91% with the second-best method, BERT (Devlin et al., 2018). This excellent classification result (**80.54%**) on this dataset also reveals the efficacy of our method. In the fifth column of Table 2, we observe that our method produces competitive results with the accuracy of 89.58% against the best accuracy of 92.52%) on our new proposed dataset (NepaliLinguistic). It outperforms one of the recent DL-based method, BERT (Devlin et al., 2018). Nevertheless, since the probability-based embedding vectors rely on the total number of tokens per category and occurrence of the input token in them, our method has been unable to achieve useful semantics from categories having a similar number of whole tokens and input token frequency per category. We notice that our dataset has several categories with a similar number of tokens and input token frequency per category compared to the other three datasets. Wherefore, we speculate that probability-based embedding vectors have diminished the performance slightly in the end.

In summary, our method outperforms all eight methods (five traditional methods and three DL-based methods) significantly on three datasets and comparable performance on the fourth dataset. Importantly, our method outperforms the BERT (Devlin et al., 2018), one of the recent DL-based methods, on all four datasets. Through this experiment, we speculate that pre-trained weights of the BERT model comprise broad categories, not just limited to the news document domain. Since it exploits the knowledge from multiple domains, it may be less effective to leverage the semantic knowledge for a specific domain such as a news document compared to our method. Also, such encouraging results further show that the domain-specific semantic relationship of tokens is very important to discriminate news documents, especially for Nepali news documents. To this end, we believe that the use of a simple probabilistic-based method can have a big role to capture the semantic information of the input token for news documents.

### Class-wise analysis

We analyze the class-wise performances of our proposed method from the classified confusion matrix. While looking at the confusion matrix for 16NepaliNews and CombinedNepaliNews, we observe that the National news category from both datasets contains intersecting information from most of the remaining categories, which exacerbates the classification performance. Similarly, while observing the confusion matrix for the NepaliNewsLarge, we notice that business and interview categories contain some intersecting information, which worsens the performance in the end. Also, while observing the confusion matrix for the NepaliLinguistic, we observe that different category pairs such as Art and Music, Filmy and Entertainment, Society and National have common information, as a result of which it diminishes the classification performance for those categories. The confusion matrices are provided in Section 8 of the Supplemental File.

### Analysis of our methods using other metrics

From the confusion matrices, we analyze the performance of our proposed method on all four datasets against other measures like Precision, Recall and *F*-score in addition to accuracy, which is calculated using confusion matrix. While observing the metrics for 16NepaliNews, we notice that our method imparts the least performance (Precision, Recall and *F*-score) among other datasets. This may be because of higher data imbalance problem in it compared to other counterpart datasets because data imbalance issue not only creates bias during training and testing but also affects during probability-based word embedding vector extraction. This is because the probability-based word embedding vector is related to the total number of tokens and their occurrence. Furthermore, few data imbalance problem, which is in both several documents and number of total tokens, has also been observed in two other datasets—NepaliNewsLarge and CombinedNepaliNews. This results in lower performance in these two datasets as well. NepaliLinguistics yields the best performance compared to other counterpart datasets because it has a comparatively balanced class distribution. However, it also has some other problems such as overlapping of tokens in several categories, imbalanced number of tokens, etc. Thus, this also attributes to lower performance against other methods in result. Refer to Section 9 of the Supplemental File for Precision, Recall, *F*-score and Accuracy of our method on four datasets. Despite such problems on all four datasets, the results show that our method has a higher Precision value.

To summarise, we speculate that our method is prominent for most of the categories on four Nepali news datasets, although it still has some problems dealing with some categories having common tokens.

### Complexity analysis

For the feature extraction of a text document using our method, we have devised five different algorithms (refer to Section 6 in the Supplemental File), where each algorithm has its computational complexity. First, Alg. 1, which is used to extract unique tokens in each document in the pre-processing step, imparts *O*(|*T*| × |*T*| × |*Z*|), where |*T*| indicates the total number of words in the document and |*Z*| is the length of the individual word of the document. The total time complexity of unique tokens calculation for all documents is the multiplication of several documents with the above time complexity. Next, Alg. 2, which is used to design the supervised codebook, relies on two main algorithms (Alg. 3 and Alg. 4) in addition to similarity calculation. Importantly, Alg. 3 imparts *O*(|*X*| × |*X*|) complexity, where |*X*| represents the total length of the corpus under the corresponding category. And, Alg. 4, which calculates the frequency of the word, imparts *O*(|*X*|) complexity. For similarity calculation, it consumes *O*(|*L*| × |*C*|), where |*L*| and |*C*| denote the total number of neighboring tokens and a total number of categories on the dataset, respectively. Overall, Alg. 2 yields *O*(|*D*| × (|*D*^i^| × |*D*^i^| × |*n*^i^| × |*n*^i^| × |*f*^i^| × |*f*^i^|) + (|*L*| × |*C*|)) complexity, where |*D*|, |*n*^i^| and |*f*^i^| denote length of corpus, length of neighboring tokens,and length of frequencies, respectively. Last, Alg. 5, which is the final step to extract the proposed features representing the document, imparts *O*(*n* × *m*) complexity, where *n* and *m* denote the length of the pre-processed document with unique tokens (|*P*|) and length of the supervised codebook (|*F*|), respectively. Note that our time complexities do not include complexities related to the SVM classifier.

## Conclusion and future works

In this article, we have proposed a novel method using the supervised codebook approach to represent the Nepali documents for the classification purpose, which can be used in several domains such as online news analysis, forecasting, etc. Extensive experimental result on four Nepali news datasets shows that our method outperforms all state-of-the-art methods on three datasets and provides competitive result on the fourth dataset. It is interesting to note that our method has outperformed the BERT-based method on all datasets. This infers the efficacy of our method against the recent DL-based method for the Nepali document representation and classification.

Our method does not require heavy computations (during both training and testing) compared to different state-of-the-art algorithms such as BERT or GloVe or Word2Vec, etc. Similarly, our method does not need heavy computational resources such as a Graphical Processing Unit (GPU). Furthermore, the embedding vector adopted in our method is very easy to compute. In contrast, since our method relies on a supervised codebook, which may be impractical sometimes as we may not be aware of the actual class labels, the performance is dependent on it. Also, the extraction of the word embedding vector based on one domain may not work on other domains such as business, health, etc.

To fulfill the gaps of our method, it would be interesting to use an unsupervised learning approach like the *k*-means algorithm for both codebook design and word embedding extraction using the training dataset. This helps to learn the features without requiring the class labels on the training dataset and may work for all domains of text documents. We also believe the dataset we have released to the public will be useful for the Nepali NLP research community.

## Supplemental Information

10.7717/peerj-cs.412/supp-1 Supplemental Information 1 Source code in Python language.

10.7717/peerj-cs.412/supp-2 Supplemental Information 2 Supplemental information.

## Additional Information and Declarations

### Data Availability

The following information was supplied regarding data availability: 16NepaliNews data is available at GitHub: https://github.com/sndsabin/Nepali-News-Classifier/blob/master/16NepaliNews.rar. NepaliNewsLarge data is available at Kaggle: https://www.kaggle.com/ashokpant/nepali-news-dataset-large. NepaliLinguistic data is available at IEEEDataPort: https://ieee-dataport.org/documents/nepaliliinguistic.
