# Supplementary material for: Vector representation based on a supervised codebook for Nepali documents classification
Source: PeerJ Comput Sci. 2021 Mar 3;7:e412. doi: 10.7717/peerj-cs.412 (PMC7959666; doi:10.7717/peerj-cs.412)
Supplement: Supplemental Information 2 [file peerj-cs-07-412-s002.pdf]

Supplementary file: Vector representation based on a supervised codebook for Nepali documents classification

Chiranjibi Sitaula[1], Anish Basnet[2] and Sunil Aryal[1]

[1]Deakin University, Geelong, Victoria, Australia
[2]Ambition College, Kathmandu, Nepal

1 INTRODUCTION

This supplementary file contains the supporting information of our work presented in the form of tables, equations, and confusion matrix. We have organized them into different sections as follows: Section 2 explains the merits and demerits of the existing methods; Section 3 provides the detailed information of our dataset; Section 4 presents the information related to Nepali texts; Section 5 mentions the supervised codebook size used in our method on four datasets; Section 6 presents the algorithms used in our methods; Section 7 enlists the train/test splits of datasets used in our work; Section 8 presents the class-wise analysis of our method on four datasets; and Section 9 lists the performance of our method based on Precision, Recall, and F-score.

2 PROS AND CONS OF EXISTING METHODS

In this section, we present the advantages and disadvantages of recent previous methods in terms of simplicity and performance. For this, we have presented into two tables (Table 1 and Table 2). Table 1 lists the advantages and disadvantages of methods based on Nepali document representation and classification tasks, whereas Table 2 presents the advantages and disadvantages of methods using non-Nepali document representation and classification tasks.

Source	Approach	Advantages	Disadvantages
Thakur and Singh (2014)	BoW+Naive Bayes	• Easy and simple to implement.<br>• Works for all types of documents.	• Limited classification performance as it is unable to deal with semantic tags.
Kafle et al. (2016)	TF-IDF+Word2Vec	• Simple and easy.<br>• Works for all types of documents.	• Provides a limited classification performance as it may not deal with semantic tags.
Singh (2018)	TF-IDF+Word2Vec + GRU	• Simple and easy to use for the experiment.	• Limited performance while using deep learning method (GRU).
Basnet and Timalsina (2018)	Word2Vec+LSTM	• Adopts sequence of tokens, which captures semantic meaning of words.	• Limited accuracy due to limited data for training the LSTM model, which seems over-fitting.<br>• Tedious to tune the optimal architecture of deep learning model (e.g., LSTM).
Shahi and Pant (2018)	TF-IDF+SVM+ANN	• Easy and simple.<br>• Works for all types of documents.	• Limited performance as they may not deal with semantic tags.
Dangol et al. (2018)	N-gram	• Shows the semantics of words using n-gram model.<br>• Improved performance than BoW method.	• The use of n-gram in their method increases computational complexity significantly and it is difficult to choose the optimal number of n.
s<br>Subba et al. (2019)	BoW+RNN	• Shows the semantics of tags using RNN.<br>• Outperforms traditional ANN.	• Difficult to tune the architecture of RNN.<br>• Provides the limited performance due to the lack of enough data.

Table 1. Advantages and disadvantages of different existing methods for Nepali document representation and classification

Source	Approach	Advantages	Disadvantages
Mourão et al. (2018)	Net-class	• Shows the semantic association of tokens.<br>• Shows that their method is computationally efficient for short texts.	• Computationally inefficient for long texts.
Kim et al. (2019)	TF-IDF+ LDA + Doc2Vec	• Improves the performance significantly.<br>• Easy to implement and use for the experiment.	• Imparts higher computational complexity for large documents.
Elnagar et al. (2020)	Deep learning (DL) algorithms (e.g. LSTM, BiLSTM, CNN, etc.)	• Identifies the appropriate DL algorithm for Arabic text classification.	• Lacks outlier tokens detection methods.
Shan et al. (2020)	Incremental Learning	• Uses reinforcement approach, which improve the performance significantly.<br>• Works for different datasets.	• Since it uses deep learning algorithm, it requires massive dataset for better feature extraction ability.
Silva et al. (2020)	BoW	• Simple to implement and analyze.	• Unable to capture the semantics of tokens.
Faustini and Covões (2020)	BoW+Word2Vec	• Simple to implement and analyze.	• Unable to show the semantic association of tokens in the document during representation, which could improve the performance.
Kim et al. (2020)	Capsule Networks	• Adopts static routing approach to minimize the computational burden in Capsule Net.<br>• Outperforms the CNNs.	• Do not adopt any techniques to show the semantic association of tokens during training of Capsule Net.
Wang et al. (2020)	CNN+BiLSTM	• Preserves token semantics using the LSTM model.<br>• Provides prominent classification accuracy.	• DL-based method always demands massive amount of data to achieve the prominent accuracy.

Table 2. Advantages and disadvantages of different existing methods for non-Nepali document representation and classification

3 DETAILED INFORMATION OF OUR DATASET

In this section, we list more detailed information of our datasets (Table 3). It contains the name of categories, number of documents and number of tokens.

Category	# of documents	# of tokens
Art	3,218	463,650
Bank	7,135	758,682
Blog	419	201,478
Business	3,282	596,952
Diaspora	195	26,565
Entertainment	1,084	202,044
Filmy	1,048	127,101
Health	162	39,761
Hollywood-bollywood	1,892	230,249
Koseli	884	485,943
Literature	1,112	266,954
Music	794	95,041
National	1,190	217,510
Opinion	1,558	6572,805
Society	3,619	505,314
Sports	6,344	894,471
World	1,715	252,905

Table 3. NepaliLinguistic dataset description

4 SAMPLE INFORMATION RELATED TO NEPALI TEXT DOCUMENTS

We present list of stop words (Table 4), list of characters (Table 5), raw and processed tags (Table 6), sample embedding vectors (Table 7), and sample codedbook (Table 8) in this section.

Stop words
हुन्थे, होलान्, थिइन्, गर्‍यो, आफै, खै, हौँ, गर्छौं, राख्छ, म, मलाई, तिमी, जो, जे.......................त्यो कि, जुन ,यी ,का...गरि ,ती, लाई,छौं

Table 4. Examples of stop words used in our method.

Pre-defined list of characters in Nepali documents						
ू , ु	ि , ी	उ , ऊ	इ , ई	ए , ऐ	ँ , ं	क , क्
ख , ख्	ग , ग्	घ , घ्	ङ , ङ्	च , च्	छ , छ्	ज , ज्
झ , झ्	ञ , ञ्	ट , ट्	ठ , ठ्	ड , ड्	ढ , ढ्	ण , ण्
त , त्	थ , थ्	द , द्	ध , ध्	न , न्	प , प्	फ , फ्
ब , ब्	भ , भ्	म , म्	य , य्	र , र्	ल , ल्	व , व्
श , श् , ष , ष् , स , स्	ह , ह्	क्ष , क्ष्	त्र , त्र्	ज्ञ , ज्ञ्		

Table 5. List of pre-defined alphabets (or characters) to be used for identifying common tokens. Note that the characters in the same cell are considered as the same in our word.

**Raw text**	१४ मंसिर, काठमाडौं । सरकारले सोमबारदेखि ड्राइभिङ लाइसेन्समा 'स्मार्ट कार्ड' प्रविधि कार्यान्वयनमा ल्याएको छ । यातायात व्यवस्था विभागले पहिलो 'स्मार्ट लाइसेन्स' सोमबार महानिर्देशक चन्द्रमान श्रेष्ठका नाममा जारी गरेको छ । उपप्रधान तथा भौतिक पूर्वाधार तथा यातायात व्यवस्था मन्त्री विजयकुमार गच्छदारले श्रेष्ठलाई पहिलो स्मार्टकार्ड हस्तान्तरण गरे । पुरानो प्रविधिको सवारी चालक अनुमतिपत्र विस्थापन गर्न आधुनिक विद्युतीय प्रविधिको स्मार्ट कार्डमा रुपान्तरण गर्न सुरु गरिएको विभागले जनाएको छ ।
**Pre-processed text**	मंसिर, काठमाडौं, सरकार, सोमबार, ड्राइभिङ, लाइसेन्स, स्मार्ट, कार्ड, प्रविधि , कार्यान्वयन, ल्याए, यातायात, व्यवस्था, विभाग, लाइसेन्स, सोमबार, महानिर्देशक , चन्द्र, श्रेष्ठ, जारी, उपप्रधान, भौतिक, पूर्वाधार, मन्त्री, विजय, गच्छदार, श्रेष्ठ, स्मार्टकार्ड, हस्तान्तरण, पुरानो, प्रविधि, सवारी, चालक, अनुमतिपत्र, विस्थापन, आधुनिक, विद्युतीय कार्ड, रुपान्तरण, जनाए

Table 6. Pre-processed text of a sample raw Nepali news document.

Word	Embedding Vector
भाषाशास्त्री	[0.00000000e+00, 0.00000000e+00, 0.00000000e+00, 0.00000000e+00, 0.00000000e+00, 0.00000000e+00, 0.00000000e+00, 8.08845535e-06, 0.00000000e+00, 0.00000000e+00, 1.87208248e-06, 3.89574974e-06, 0.00000000e+00, 0.00000000e+00, 0.00000000e+00, 0.00000000e+00]
रकम	[6.54266754e-04, 2.88411763e-03, 2.79095730e-04, 7.52477497e-04, 1.69345720e-03, 1.49231458e-04, 2.10184010e-03, 3.55892035e-04, 1.36187830e-04, 9.85512959e-05, 5.94386186e-04, 3.46721727e-04, 5.36122051e-04, 7.15705765e-04, 5.00307882e-04, 3.66283595e-04]
जागीर	[0.00000000e+00, 0.00000000e+00, 1.67457438e-04, 0.00000000e+00, 0.00000000e+00, 0.00000000e+00, 0.00000000e+00, 1.61769107e-05, 0.00000000e+00, 0.00000000e+00, 9.36041238e-06, 7.79149947e-06, 6.61879075e-06, 0.00000000e+00, 0.00000000e+00, 0.00000000e+00]

Table 7. Probability-based word embedding vectors of three tokens in the 16NepaliNews dataset.

Sample supervised codebook
[प्राधिकरण , प्रविधि , विगत , उत्पन्न, ब्राण्ड, विषय, कहिले, छिमेकी, दल, सेवाको, व्यवहार, गते, तिर्न, सल्लाह , प्राथमिकता............... एमाओवादी...कार्यकर्ता]

Table 8. Supervised codebook extracted from 16NepaliNews dataset.

Algorithm 1 UNIQUE_TOKENS(T,C)

Input: $T \leftarrow$ Set of tokens, $C \leftarrow$ Pre-defined list of special alphabets
Output: $P \leftarrow \{\}$ {Unique tokens}

```
for i = 0 to |T| do
    for j = 0 to |T| do
        Z ← GET_LETTERS(T[i]) {Get characters of first token}
        S ← GET_LETTERS(T[j]) {Get characters of second token}
        if LEN(Z) == LEN(S) then
            for k = 0 to |Z| do
                if Z[k]! = S[k]&(Z[k]ANDS[k]) ∈ C then
                    T.Remove(T[j]) {Remove and update the length of list}
                end if
            end for
        end if
    end for
end for
P ← T {Assign the resultant output to P}
return P
```

5 SAMPLE CODEBOOK SIZE ON FOUR DATASETS

In this section, we present the codebook size used in our method for all the datasets (Table 9). Since we have used five folds in our method, we have listed codebook size used for all five folds of each dataset in the table.

Dataset	Set 1	Set 2	Set 3	Set 4	Set 5
16NepaliNews	319	309	320	329	319
NepaliNewsLarge	285	237	250	247	246
CombinedNepaliNews	328	340	345	346	333
NepaliLinguistics	582	527	506	520	572

Table 9. Supervised codebook size extracted for all sets on the corrresponding dataset.

6 ALGORITHMS USED IN OUR PROPOSED METHOD

In this section, we present the list of algorithms used in our method. We have adopted five different algorithms (Algs. 1, 2, 3, 4, and 5). Specifically, Alg. 1 is used to extract the unique tokens present in the document during the pre-processing stage. Similarly, Alg. 2 provides the supervised codebook using training corpus. Moreover, Algs. 3 and 4 extracts the neighboring tokens and frequency of tokens in the document, respectively. And Alg. 5 presents the step-wise procedure to represent the document.

Algorithm 2 Design supervised codebook using training corpus

```
Input: C ← {C^1, C^2, ··· , C^p} {Class labels},
    D ← {D^1, D^2, ··· , D^p} {Corpus under corresponding classes or categories}
Output: F ←[]{Supervised codebook}
    {Calculate neighbours and frequency}
for i = 0 to |D| do
    L ← []
    n^i ← GET_NEIGHBOURS(C^i,D^i)
    for k = 0 to |n^i| do
        f^i ← GET_FREQ(n^i[k],D^i)
        for j = 0 to f^i do
            if f^i[j]>GET_FREQ(C^i,D^i) then
                L.Append(n^i[j])
            end if
        end for
    end for
    {Words ranking in the documents}
    for l = 0 to |L| do
        x ← cos(L[l],C^i)
        S ← []
        for n = 0 to |C| do
            S.Append(cos(L[l),C^n))
        end for
        y ← MAX(S) {Calculate maximum similarity value}
        if x>y then
            F.Append(L[l])
        end if
    end for
end for
return F
```

Algorithm 3 Calculate GET_NEIGHBOURS(W, X)

```
Input: W ← Root word to search for its neighbours, X ← Corpus to be used for searching neighbours of
    W
Output: L ← []{List of neighbours}
for i = 0 to |X| do
    for k = 0 to |X[i]| do
        if t == X[i][k] then
            if k+1! = |X[i][k] − 1| AND k − 1! = 0 then
                L.Append(X[i][k − 1])
                L.Append(X[i][k + 1])
            else if k + 1 == |X[i][k] − 1| then
                L.Append(X[i][k − 1])
            else
                L.Append(X[i][k + 1])
            end if
        end if
    end for
end for
return L
```

Algorithm 4 Calculate GET_FREQ(W, X)

Input: $W \leftarrow$ Word to be searched , $X \leftarrow$ Corpus from where we extract the frequency of the word W
Output: $A \leftarrow$ Frequency of W in X

for $i = 0$ to $|X|$ **do**
  $A = 0$
  if $t \in X[i]$ **then**
    $A++$
  end if
end for
return A

Algorithm 5 Proposed features extraction method

Input: $P \leftarrow$ Pre-processed document, $F \leftarrow$ Supervised codebook
Output: $P(S) \leftarrow []$

$T \leftarrow []$
{Module for generating document matrix of P}
for $i = 0$ to n **do**
  $t \leftarrow []$
  for $j = 0$ to m **do**
    $s \leftarrow$ cos(P[i],F[j])
    t.Append(s)
  end for
  T.Append(t)
end for
{Module for average pooling in the matrix T}
for $j = 0$ to m **do**
  $SUM = 0$
  for $i = 0$ to n **do**
    $SUM += T_j^i$
  end for
  $P(S_j) \leftarrow \frac{Sum}{n}$
end for
return $P(S)$

7 DETAILED INFORMATION OF DATASETS

In this section, we first present the detailed information for each dataset and also the train/test split of each of them. First, we explain each dataset which elaborates the name and other details.

16NepaliNews contains 14,364 under 16 categories, where each category contains at least 16 documents. The names of categories in this dataset are Auto, Bank, Blog, Business Interview, Economy, Education, Employment, Entertainment, Interview, Literature, National News, Opinion, Sports, Technology, Tourism, and World.

NepaliNewsLarge contains 7,023 document under 20 news categories, where each category contains 111 to 700 documents. The names of categories in this dataset are Agriculture, Automobiles, Bank, Blog, Business, Economy, Education, Employment, Entertainment, Health, Interview, Literature, Migration, Opinion, Politics, Society, Sports, Technology, Tourism, and World.

CombinedNepaliNews contains 21,387 document under 21 categories, where each category contains 111 to 7,452 documents. We design this dataset by the combination of two publicly datasets: NepaliNewsLarge and 16NepaliNews. The names of categories in this dataset are Agriculture, Auto, Bank, Blog, Business, Economy, Education, Employment, Entertainment, Health, Interview, Literature, Migration, National News, Opinion, Politics, Society, Sports, Technology, Tourism, and World.

NepaliLinguistic, which is a new dataset we prepared and will be made publicly available, contains 17 news categories. This dataset contains 35,651 documents in total, where each category contains at least 67 documents. The names of categories in this dataset are Art, Bank, Blog, Business, Diaspora, Entertainment, Filmy, Health, Hollywood-bollywood, Koseli, Literature, Music, National, Opinion, Society, Sports, and World.

Second, we present the number of train/test split of each dataset (Table 10). Also, we present the total number of documents in the table. This statistics help learn the distribution of documents in each dataset.

Dataset	Train	Test	Total
16NepaliNews	12,920	1,444	14,364
NepaliNewsLarge	6,309	714	7,023
CombinedNepaliNews	19,242	2,145	21,387
NepaliLinguistic	32,078	3,573	35,651

Table 10. Dataset description

8 CLASS-WISE ANALYSIS OF OUR METHOD USING CONFUSION MATRIX

In this section, we present four representative confusion matrix achieved from each of four datasets (Figs. 1 for D1, 4 for D2, 2 for D3, and 3 for D4).

Predicted

Actual

	Auto	Bank	Blog	Business Interview	Economy	Education	Employment	Entertainment	Interview	Literature	National News	Opinion	Sports	Technology	Tourism	World
Auto	3	0	0	0	1	0	0	0	0	0	6	0	0	0	0	0
Bank	0	21	0	0	7	0	0	0	0	0	6	0	0	0	0	0
Blog	0	0	0	0	0	0	0	1	0	0	10	10	0	0	0	0
Business Interview	0	0	0	0	4	0	0	0	0	0	11	0	0	0	0	0
Economy	0	5	0	0	63	0	0	0	0	0	50	0	0	0	0	0
Education	0	0	0	0	0	1	0	0	0	0	9	0	0	0	0	0
Employment	0	0	0	0	5	0	2	0	0	0	9	0	0	0	0	0
Entertainment	0	0	0	0	0	0	0	100	0	0	14	0	1	0	0	0
Interview	0	0	0	0	0	0	0	6	0	0	2	1	0	0	0	0
Literature	0	0	0	0	0	0	0	0	0	2	0	0	0	0	0	0
National News	0	0	0	0	7	0	0	1	0	0	730	7	1	0	0	0
Opinion	0	0	0	0	0	0	0	0	0	1	24	35	0	0	0	0
Sports	0	0	0	0	0	0	0	0	0	0	23	0	210	0	0	0
Technology	0	0	0	0	0	0	0	0	0	0	6	0	0	6	0	0
Tourism	0	0	0	0	5	0	0	0	0	0	14	0	0	0	3	0
World	0	0	0	0	0	0	0	0	0	0	16	0	0	0	0	6

Figure 1. Confusion matrix on the testing set of 16NepaliNews dataset (Set 1)

Predicted

Actual

	Agriculture	Automobiles	Bank	Blog	Business	Economy	Education	Employment	Entertainment	Health	Interview	Literature	Migration	Opinion	Politics	Society	Sports	Technology	Tourism	World
Agriculture	12	0	0	0	0	6	0	0	0	1	0	0	0	0	0	0	0	0	0	0
Automobiles	0	21	0	0	1	1	0	0	1	0	1	0	0	0	0	0	0	0	0	1
Bank	0	1	50	0	0	6	0	1	1	0	0	0	1	1	0	0	0	1	0	0
Blog	0	0	0	2	0	2	0	0	2	0	1	3	0	10	0	0	6	0	0	0
Business	1	0	1	0	18	0	0	0	0	0	10	0	0	0	0	0	1	0	0	0
Economy	4	4	4	0	3	34	0	1	1	0	0	0	0	1	0	1	1	1	0	5
Education	0	0	0	0	0	1	13	0	2	0	1	1	0	0	0	1	0	0	0	0
Employment	1	0	1	0	1	5	1	17	2	0	0	0	0	1	2	0	0	0	0	0
Entertainment	0	0	0	0	0	4	1	0	50	0	0	4	0	1	1	2	1	0	0	0
Health	0	0	0	0	0	1	0	0	0	14	0	0	0	2	1	0	0	0	0	0
Interview	0	0	0	0	12	1	0	1	1	0	16	0	0	2	0	0	1	0	0	0
Literature	0	0	0	0	0	0	0	0	1	0	0	18	0	7	0	1	0	0	0	0
Migration	0	0	0	0	0	1	0	1	2	0	0	0	4	0	0	2	1	0	1	0
Opinion	0	0	0	0	0	0	0	0	0	0	1	0	0	49	0	0	0	0	0	0
Politics	0	0	0	0	0	0	0	0	2	0	0	0	0	0	52	0	0	0	0	1
Society	0	0	0	0	0	4	0	0	3	2	0	0	0	1	3	22	0	0	1	0
Sports	0	0	0	2	0	2	0	0	1	0	0	0	0	0	1	2	60	0	1	1
Technology	0	0	0	0	1	3	0	0	0	0	2	0	0	1	0	0	0	5	0	0
Tourism	0	0	0	0	0	5	0	0	0	0	0	0	0	0	1	0	0	0	21	0
World	0	0	0	0	0	5	0	0	5	0	0	2	0	0	3	2	0	1	0	14

Figure 2. Confusion matrix on the testing set of NepaliNewsLarge dataset (Set 3).

	Predicted																				
**Actual**	Agriculture	Auto	Bank	Blog	Business	Economy	Education	Employment	Entertainment	Health	Interview	Literature	Migration	National News	Opinion	Politics	Society	Sports	Technology	Tourism	World
Agriculture	16	s0	0	0	0	1	0	0	0	0	0	0	0	3	0	0	0	0	0	0	0
Auto	0	33	0	0	0	0	0	0	0	0	0	0	0	2	0	0	0	0	0	0	0
Bank	0	0	86	0	0	1	0	0	0	0	0	0	0	7	0	0	2	0	0	1	0
Blog	0	0	0	42	0	0	0	0	1	0	0	0	0	2	0	0	0	2	0	0	0
Business	0	0	3	0	31	4	0	1	0	0	5	0	0	1	0	0	0	0	0	0	0
Economy	0	4	7	0	3	130	1	2	1	0	0	0	0	28	0	0	0	0	0	1	0
Education	0	0	0	0	0	0	18	0	0	0	0	0	0	9	0	0	0	0	0	0	0
Employment	0	0	0	0	0	2	0	38	0	0	0	0	0	6	0	0	0	0	0	0	0
Entertainment	0	0	0	0	0	0	0	0	160	0	1	0	0	17	0	0	0	4	0	0	0
Health	0	0	1	0	0	0	0	0	0	13	0	0	0	3	1	0	0	0	0	0	0
Interview	0	0	0	0	19	0	0	0	0	0	18	0	0	2	3	0	0	0	0	0	0
Literature	0	0	0	0	0	0	0	0	2	0	0	17	0	8	0	0	0	0	0	0	0
Migration	0	0	0	0	0	1	0	0	0	0	0	0	3	8	0	0	0	0	0	0	0
National News	0	0	6	1	0	28	2	4	7	1	0	1	0	660	4	0	3	20	0	3	1
Opinion	0	0	0	0	0	0	0	0	0	0	0	0	0	6	102	0	0	0	0	0	0
Politics	0	0	0	0	0	0	0	0	0	0	0	0	0	54	0	0	0	0	0	1	0
Society	0	0	0	0	0	0	1	0	0	0	0	0	0	22	0	0	11	2	0	0	0
Sports	0	0	0	3	0	1	0	0	3	0	0	0	0	36	0	0	2	250	0	0	0
Technology	0	0	0	0	0	0	0	0	0	0	0	0	0	0	0	0	0	0	23	0	0
Tourism	0	0	0	0	0	0	0	0	0	0	0	0	0	4	0	0	0	0	0	44	0
World	0	0	0	0	0	5	0	0	2	0	0	0	0	5	0	0	0	0	0	0	41

Figure 3. Confusion matrix on the testing set of CombinedNepaliNews dataset (Set 5).

Predicted

Actual	Art	Bank	Blog	Business	Diaspora	Entertainment	Filmy	Health	Hollywood-bollywood	Koseli	Literature	Music	National	Opinion	Society	Sports	World
Art	290	0	0	4	0	7	1	0	1	2	2	0	2	0	10	1	2
Bank	0	710	1	0	0	0	0	0	0	0	1	4	0	0	0	0	0
Blog	0	1	30	0	0	0	0	0	0	2	7	0	0	1	1	0	0
Business	0	0	0	310	0	0	0	0	0	2	0	0	5	6	4	2	3
Diaspora	0	0	0	3	5	1	0	0	0	0	0	0	1	0	1	2	7
Entertainment	13	0	0	2	0	82	0	0	0	6	0	0	2	0	3	0	1
Filmy	2	1	0	0	0	0	80	0	4	0	3	15	0	0	0	0	0
Health	0	0	0	0	0	0	0	13	0	0	0	0	1	2	0	0	1
Hollywood-bollywood	4	0	0	0	0	0	1	0	180	0	0	2	0	0	0	0	0
Koseli	0	0	0	1	0	2	0	0	0	71	0	0	1	6	0	8	0
Literature	2	2	1	0	0	0	1	0	0	2	100	0	0	1	0	0	0
Music	0	2	0	0	0	0	12	0	3	0	3	60	0	0	0	0	0
National	1	0	0	3	0	2	0	1	0	0	0	0	93	2	16	0	1
Opinion	0	0	0	5	0	0	0	0	0	3	0	0	0	150	1	0	0
Society	6	2	0	9	0	0	0	1	0	0	0	0	19	1	320	1	0
Sports	1	0	0	0	0	0	0	0	0	0	0	0	0	0	1	630	1
World	3	0	0	7	0	0	0	1	0	3	0	0	2	0	3	0	150

Figure 4. Confusion matrix on the testing set of NepaliLinguistic (Set 1).

9 ANALYSIS OF PROPOSED METHOD ON FOUR DATASETS

In this section, we present the overall analysis result of four dataset using Precision, Recall, and F-score (Table 11). This result helps understand the efficacy of our method using such metrics. Similarly, we present the formula of such metrics in Eqs. (1) for Precision, (2) for Recall, (3) for F-score, and (4) for Accuracy.

Dataset	Precision	Recall	F-score
16NepaliNews	**64.20**	40.60	46.40
NepaliNewsLarge	**69.60**	61.80	63.00
CombinedNepaliNews	**80.20**	68.40	72.00
NepailLinguistic	**83.60**	79.00	80.60

Table 11. Average performance based on micro-averaged Precision, Recall, and F-score of all splits on four datasets.

$$\text{Precision} = \frac{TP}{TP+FP}, \tag{1}$$

$$\text{Recall} = \frac{TP}{TP+FN}, \tag{2}$$

$$\text{F-score} = 2 \times \frac{(\text{Recall} \times \text{Precision})}{(\text{Recall} + \text{Precision})}, \tag{3}$$

$$\text{Accuracy} = \frac{TP+TN}{TP+TN+FP+FN}, \tag{4}$$

where FP, TP, FN, TN denote false positive, true positive, false negative, and true negative, respectively.

REFERENCES

Basnet, A. and Timalsina, A. K. (2018). Improving nepali news recommendation using classification based on lstm recurrent neural networks. In *Proc. International Conference on Computing, Communication and Security (ICCCS)*, pages 138–142.

Dangol, D., Shrestha, R. D., and Timalsina, A. (2018). Automated news classification using n-gram model and key features of nepali language. *SCITECH Nepal*, 13(1):64–69.

Elnagar, A., Al-Debsi, R., and Einea, O. (2020). Arabic text classification using deep learning models. *Information Processing & Management*, 57(1):102121.

Faustini, P. H. A. and Covões, T. F. (2020). Fake news detection in multiple platforms and languages. *Expert Systems with Applications*, page 113503.

Kafle, K., Sharma, D., Subedi, A., and Timalsina, A. K. (2016). Improving nepali document classification by neural network. In *Proc. IOE Graduate Conference*, pages 317–322.

Kim, D., Seo, D., Cho, S., and Kang, P. (2019). Multi-co-training for document classification using various document representations: Tf–idf, lda, and doc2vec. *Information Sciences*, 477:15–29.

Kim, J., Jang, S., Park, E., and Choi, S. (2020). Text classification using capsules. *Neurocomputing*, 376:214–221.

Mourão, F., Rocha, L., Viegas, F., Salles, T., Gonçalves, M., Parthasarathy, S., and Meira Jr, W. (2018). Netclass: A network-based relational model for document classification. *Information Sciences*, 469:60–78.

Shahi, T. B. and Pant, A. K. (2018). Nepali news classification using naïve bayes, support vector machines and neural networks. In *Proc. International Conference on Communication Information and Computing Technology (ICCICT)*, pages 1–5.

Shan, G., Xu, S., Yang, L., Jia, S., and Xiang, Y. (2020). Learn#: a novel incremental learning method for text classification. *Expert Systems with Applications*, 147:113198.

Silva, R. M., Santos, R. L., Almeida, T. A., and Pardo, T. A. (2020). Towards automatically filtering fake news in portuguese. *Expert Systems with Applications*, 146:113199.

Singh, O. M. (2018). Nepali multi-class text classification. `https://oya163.github.io/assets/resume/Nepali_Text_Classification.pdf`.

Subba, S., Paudel, N., and Shahi, T. B. (2019). Nepali text document classification using deep neural network. *Tribhuvan University Journal*, 33(1):11–22.

Thakur, S. K. and Singh, V. K. (2014). A lexicon pool augmented naive bayes classifier for nepali text. In *Proc. Seventh International Conference on Contemporary Computing (IC3)*, pages 542–546.

Wang, M., Cai, Q., Wang, L., Li, J., and Wang, X. (2020). Chinese news text classification based on attention-based cnn-bilstm. In *Proc. MIPPR*, page 114300K.
